# Neuroprotective Potential of Non-Digestible Oligosaccharides: An Overview of Experimental Evidence

**DOI:** 10.3389/fphar.2021.712531

**Published:** 2021-08-23

**Authors:** Gangaraju Divyashri, Bindu Sadanandan, Kotamballi N Chidambara Murthy, Kalidas Shetty, Kumari Mamta

**Affiliations:** ^1^ Department of Biotechnology, M S Ramaiah Institute of Technology, Bengaluru, India; ^2^ Central Research Laboratory and Division of Research and Patents, Ramaiah Medical College and Hospital, Bengaluru, India; ^3^ Department of Plant Science, North Dakota State University, Fargo, ND, United States

**Keywords:** free radical, oxidative stress, prebiotics, non-digestible oligosaccharides, neuromodulation, neuroprotection

## Abstract

Non-digestible oligosaccharides (NDOs) from dietary sources have the potential as prebiotics for neuroprotection. Globally, diverse populations suffering from one or the other forms of neurodegenerative disorders are on the rise, and NDOs have the potential as supportive complementary therapeutic options against these oxidative-linked disorders. Elevated levels of free radicals cause oxidative damage to biological molecules like proteins, lipids, and nucleic acids associated with various neurological disorders. Therefore, investigating the therapeutic or prophylactic potential of prebiotic bioactive molecules such as NDOs as supplements for brain and cognitive health has merits. Few prebiotic NDOs have shown promise as persuasive therapeutic solutions to counter oxidative stress by neutralizing free radicals directly or indirectly. Furthermore, they are also known to modulate through brain-derived neurotrophic factors through direct and indirect mechanisms conferring neuroprotective and neuromodulating benefits. Specifically, NDOs such as fructo-oligosaccharides, xylo-oligosaccharides, isomalto-oligosaccharides, manno-oligosaccharides, pectic-oligosaccharides, and similar oligosaccharides positively influence the overall health *via* various mechanisms. Increasing evidence has suggested that the beneficial role of such prebiotic NDOs is not only directed towards the colon but also distal organs including the brain. Despite the wide applications of these classes of NDOs as health supplements, there is limited understanding of the possible role of these NDOs as neuroprotective therapeutics. This review provides important insights into prebiotic NDOs, their source, and production with special emphasis on existing direct and indirect evidence of their therapeutic potential in neuroprotection.

## Introduction to Prebiotics and Its Types

A balanced diet with good nutrition and physical exercise confers beneficial effects on human health and wellness. On the other hand, unhealthy lifestyle and environmental stresses can lead to several associated challenges like the onset of various diseases including mental disorders. Currently, mental disorders affect around 450 million people worldwide, causing neuro-cognitive breakdowns that are the leading causes of poor health (WHO, 2019). Enhanced levels of reactive oxygen species (ROS) and insufficient antioxidant defense mechanisms to counter them have been associated with the pathogenesis of various mental disorders including anxiety, depression, schizophrenia, Parkinson’s disease (PD), Alzheimer’s disease (AD), and many others (Manoharan et al., 2016). Prebiotics are a group of non-digestible oligosaccharides (NDOs) that are biotransformed by beneficial colonic microorganisms (probiotics) with potential health benefits to the host. Probiotics are live microorganisms which, when administered in adequate amounts, confer health benefits on the host. These gut-residing probiotics support the host health by offering resistance to pathogens, regulating the immune system, modifying insulin resistance and metabolic profile (Kelly et al., 2015), and also influencing behavioral and neurological functions (Hansan and Yang 2019). Prebiotics are widely known to modulate probiotics in humans and animals, with an overall beneficial impact on health. Although the focus of the use of prebiotics was initially towards treating digestive ailments over the last decade, the efficacy of prebiotics to suppress ROS and to positively influence mental health *via* modulation of gut-residing probiotics and through impact on the gutbrain axis is also documented (Ansari et al., 2020). Nutritional modulation of gut probiotics by prebiotics results in the formation of key beneficial metabolites such as short-chain fatty acids-SCFA, and inflammatory and immune markers which potentially offer “distal” health benefits to the brain (Cerdo et al., 2019). However, the challenge with understanding the possible role of these prebiotic functional components in brain development and function is the lack of clarity on the metabolic function and benefits of the interaction of the intestinal microbiota with the central nervous system (CNS). Considering the therapeutic benefits and applications of prebiotics, it is important to understand their neuroprotective function to integrate them into health solutions and overall wellness.

Definitions of prebiotics and global demand: Prebiotic oligosaccharides are microbiota-modulating compounds as they can serve as a carbon source that supports the growth of probiotics, thereby conferring specific or selective change in the gut to support host health *via* improvements in metabolic functions (Carlson et al., 2018). There have been several studies to develop prebiotics towards improving human health. With the increased demand for healthier foods, the interest in prebiotics has grown. The idiom “prebiotics” has caused some discrepancy and confusion among consumers worldwide. The term “prebiotics” was coined by Gibson and Roberfroid in 1995 as “non-digestible food ingredients that can be useful, affecting the host by selectively stimulating the growth and/or activity of one or more limited number of bacteria in the colon, thus improving host health (Gibson and Roberfroid 1995). In line with this definition, only a few compounds of the carbohydrate group mainly oligosaccharides, *viz.*, fructo-oligosaccharides (FOS), galacto-oligosaccharides (GOS), manno-oligosaccharides (MO), xylo-oligosaccharides (XOS) are classified as potential prebiotics (Davani-Davari et al., 2019). Since then many scientific definitions of prebiotics have evolved. The present scientific definition of prebiotics was presented in 2017 at the International Scientific Association for Prebiotics and Probiotics (ISAPP) by a panel of experts from the domains of microbiology, nutrition, and clinical research (Gibson et al., 2017). The panel-defined prebiotics as a substrate that is selectively utilized by host microorganisms conferring a health benefit (Scott et al., 2020). Thus, according to the above scientific consensus, for any compound to be called a prebiotic, it should act as a substrate for health-promoting gut microorganisms and must possess a physiological effect benefiting the host. In addition to oligosaccharide-based prebiotics, dietary fibers such as resistant starch, inulin, pectin, and beta-glucans also fit the definition of prebiotics. This updated definition paves the way for deepening our perception and understanding of prebiotics.

Regulatory agencies across the globe have their own definitions for prebiotics. Almost all the regulatory and scientific definitions consider prebiotics as dietary fibers (Table 1). However, there is more than one prebiotic that will not fall under the dietary fiber category (Carlson et al., 2018). The U.S. Food and Drug Administration (USFDA) and Food and Agriculture Organization/World Health Organization (FAO/WHO) clearly distinguish between prebiotic dietary fiber and other qualifying prebiotic compounds. The US-FDA defines the prebiotic dietary fiber as isolated or synthetic non-digestible soluble or insoluble carbohydrates with monomeric units ≥ 3, possessing physiologic effects that are beneficial to human health. The FAO/WHO states prebiotic dietary fibers as carbohydrate polymers comprising monomeric units (10 or more) that resist hydrolysis in the human small intestine by the endogenous enzymes. On the other hand, the US-FDA allows a biologically based group of foods conferring health benefits on the host to qualify as prebiotic compounds. On similar lines, the FAO/WHO defines other qualifying compounds as prebiotics when any non-viable food component results in conferring health benefits associated with microbiota modulation. Conversely, other regulatory agencies across the globe, *viz.*, the Food for Specified Health Use (FOSHU), European Food Safety Authority (EFSA), Health Canada, and Food Safety and Standards Association of India (FSSAI), have a general definition for prebiotics (Table 1). These definitions make a point that not all prebiotics are carbohydrates and all dietary fibers are prebiotics (Davani-Davari et al., 2019). The criteria used for categorizing a compound as prebiotics are that it should be resistant to the stomach’s acidic pH, resistant to hydrolysis by mammalian enzymes, can be fermented selectively by the intestinal microbiota, promote the activity, and/or growth of the microbes present in the intestine. A prebiotic fiber should possess a degree of polymerization equal to or higher than 3 (Davani-Davari et al., 2019).

**TABLE 1:** Regulatory definitions of prebiotic dietary fiber and other qualifying compounds.

Regulatory agency	Definition of Prebiotic Dietary Fiber	Definition of other qualifying compounds as Prebiotics
United States-Food and Drug Administration (US-FDA)	Synthetic or isolated non-digestible (soluble or insoluble) carbohydrates made up of monomeric units ≥ 3, and possessing physiologic effects that are beneficial to human health	Biologically-based group of foods conferring health benefits on the host
Food and Agriculture Organization/World Health Organization (FAO/WHO)	Carbohydrate polymers comprising of monomeric units (10 or more) that resist hydrolysis by the endogenous enzymes that naturally occur in the human small intestine	Non-viable food components that confer health benefits on the host by modulating beneficial gut microbiota
Food for Specified Health Use (FOSHU), Japan	Principal food ingredient that is officially approved to claim its physiological effects on human body (Brown well et al., 2012)	
European Food Safety Authority (EFSA)	Non-viable food ingredient that confers health benefits to host by modulating the gut microbiota (Definition adapted from the FAO/WHO)	
Health Canada	Term “prebiotic” is allowed only for products that are required for an approved health claim	
Food Safety and Standards Association of India (FSSAI), India	Non-viable food ingredient that provides health benefits to host by modulating the gut microbiota (Definition adapted from the FAO/WHO)	

It has been difficult to assess and measure the global consumption of prebiotics since they are found in varied food groups from natural sources, *viz.*, vegetables, fruits, milk, and honey, to wide ranges of supplements (de Souza Aquino et al., 2017; Carlson et al., 2018). The prebiotics from natural sources include resistant starch, GOS, FOS, XOS, POS, and other oligosaccharides (de Souza Aquino et al., 2017). Without the inclusive list of food ingredients on the food package, epidemiologic tracking of prebiotic consumption pattern is difficult to ascertain (Carlson et al., 2018). However, growing awareness of gut–brain connection and the consequential focus on maintaining gut health have enhanced the demand and the need for bioactive prebiotics (Hwang and Lee, 2019). Worldwide, there is a substantial growth in prebiotics demand, and this is anticipated to grow, beyond $7.5 billion by 2023 (Carlson et al., 2018). Currently, there are no authorized dietary recommendations for the adequate intake of prebiotics in healthy individuals. To maintain gut health, most prebiotics require an average oral dose of 3–5 g/day. However, daily dose recommendations depend on the nature of the food containing a prebiotic compound whether it is naturally present or specifically added (de Souza Aquino et al., 2017). Scientific evidence indicates that the consumption of inulin (5–8 g/day), FOS (3 g/day), and GOS (4 g/day) led to a significant increase in fecal probiotic bifidobacteria (de Souza Aquino et al., 2017).

Occurrence: Prebiotics exist naturally in several foods and can also be synthesized commercially using various enzymes or substrates (Lockyer and Stanner, 2019). Presently, a number of prebiotics are being investigated, and majority of them belong to a carbohydrate group and are generally oligosaccharide carbohydrates (Davani-Davari et al., 2019). These oligosaccharide carbohydrates include FOS, GOS, IMO, MO, POS, XOS, and chitin oligosaccharides (COS). FOS and GOS are most in demand on the global market currently. Published literature mostly discusses non-digestible oligosaccharide carbohydrates (NDOs) and polysaccharide prebiotics, *viz.*, inulin and resistant starch, which is well established to possess prebiotic activity. There are reports on the effects of NDOs and polysaccharide prebiotics on gut microbiota modulation (Dahiya et al., 2017; Gibson et al., 2017). Inulin is shown to counter the detrimental effects of high-fat diets on the mucus layer penetrability and metabolic functions (Schroeder et al., 2018). The mixture of FOS and GOS was able to modulate bifidobacteria by suppressing *Clostridium* levels in the gut whereas GOS alone enhanced *Lactobacillus* levels (Vandenplas, Zakharova and Dmitrieva, 2015). In addition, there is also some evidence for disaccharides such as lactulose and lactitol as potent prebiotics. Lactulose, an isomer of lactose, was shown to modulate gut microbiota by stimulating the growth of beneficial microorganisms. Gibson et al. (2010) synthesized lactulose-derived GOS, and lactulose was demonstrated to improve the quality of human life suffering from hepatic encephalopathy (Shukla et al., 2011). Investigations on evaluating the neuroprotective therapeutic mechanism of lactulose are also highlighted in the sections below (Lee et al., 2021). This review discusses prebiotic NDOs production in the introduction section, and their probable mechanisms/routes through which they offer mental health protection are highlighted in the subsequent section. Evidences in rodent models and humans are explained by the observed therapeutic effects of prebiotics. The probable mechanism through which neuroprotection effect is observed is highlighted in a separate section. The last section identifies the obvious gaps in current knowledge and avenues for future investigations.

### Prebiotic Oligosaccharide Carbohydrates

**FIGURE 1:**
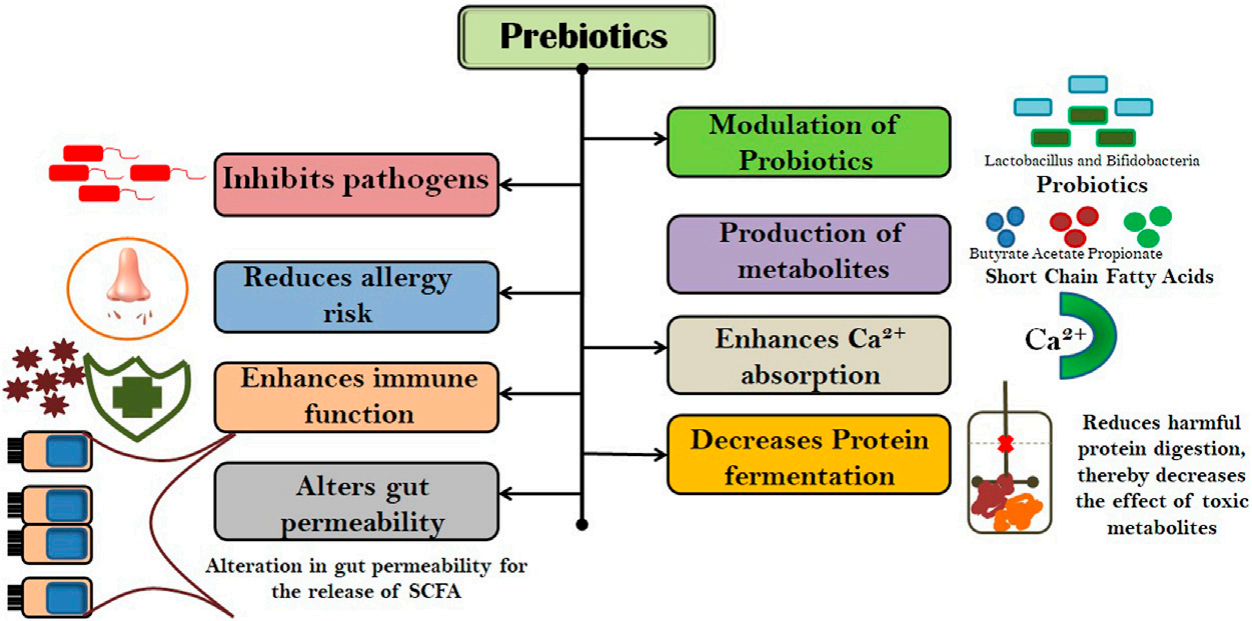
Health attributes of prebiotic NDOs.

Prebiotic oligosaccharides are carbohydrates that are chemically stable at a wide range of temperatures and pH and are classified as non-digestible oligosaccharides (NDOs) (Singh et al., 2017). Health attributes of prebiotic NDOs have been extensively reviewed and are accepted worldwide (Figure 1). However, these effects have been primarily observed towards the colon, but evidence indicates that the NDO prebiotics have the ability to modulate beyond the GIT. Beneficial gut microorganisms, *viz.*, *Lactobacillus* sp. and *Bifidobacterium* sp. selectively ferment NDOs, thereby producing a wide range of metabolites in the gut, *for example*, short-chain fatty acids (SCFAs), including butyric acid, acetic acid, and propionic acid. (Hutkins et al., 2016; Davani-Davari et al., 2019). These SCFAs (straight or branched volatile fatty acids) as metabolic products are reported to have beneficial effects on the human body (Singh et al., 2017). Acetic acid (C2) is a key metabolite in the ability of bifidobacteria to inhibit gut-related pathogens (Rios-Covian et al., 2016). Treatment for subcutaneous adipose tissue with propionic acid (C3) results in the downregulation of macrophage markers (CD163 and MMP-9) and inflammatory parameters, *viz.*, TNF-α and IP-10 (Stinson et al., 2017; Rezaee, 2019). Butyric acid (C4) is reported to alter bacterial adhesion to the gut wall by increasing mucus production (Jung et al., 2015). Therefore, SCFA appears to be very important in maintaining gut barrier function and acts as a mediator in the link between nutrition, gut microbiota, and human physiology. A study by Clarke et al. (2010) showed that prebiotic fermentation also produces peptidoglycan that enhances the innate immune system *via* downregulating bone-marrow-derived neutrophils against *Staphylococcus aureus* and *Streptococcus pneumoniae*. Thus, the effect of prebiotics on human health is mediated through their fermentation products in the gut.

Fructo-oligosaccharides (FOS): FOS occur naturally in various plant sources including onion, asparagus, *Jerusalem artichokes*, and wheat. However, FOS concentration in these sources is not sufficient to offer prebiotic activity, and therefore, there is a need for its synthesis to provide a higher dose. FOS are commercially produced using 2 processing methods: 1) batch production of FOS using sucrose as a substrate and employing fructosyltransferase (FTase) and 2) continuous production using sucrose as a substrate and immobilized FTase (Ashwini et al., 2019). Several microbes produce significant titers of FTase. These include species in the genera of *Aspergillus*, *Aureobasidium*, *Penicillium*, and *Fusarium* (Davani-Davari et al., 2019). FOS produced from FTase are a mixture of oligosaccharides with a degree of polymerization (DP) 3–5. Structurally, FOS consist of fructose units linked to the terminal glucose *via* β-(1, 2) linkages. Galacto-oligosaccharides (GOS) are commercially produced from lactose by β-galactosidase. However, previous attempts in commercial production of GOS using galactosyl-transferase were not very successful and economical because galactosyl-transferase is more stereoselective than β-galactosidase, and it requires nucleotide sugars as the donor (Davani-Davari et al., 2019). A wide range of microorganisms (bacteria, fungi, and yeast) produce β-galactosidase, and this affects GOS production in terms of the degree of polymerization (DP), amount of GOS produced, and glycosidic linkages (Osman et al., 2012). Structurally, GOS consist of oligosaccharides mixture comprising of galactose units linked to a terminal glucose moiety with DP ranging from 3 to 8 linked *via.*, β (1–6), β (1–3), and α (1–4) linkages (Torres et al., 2010; Vera et al., 2016). Xylo-oligosaccharides (XOS): XOS occur naturally in honey, bamboo shoots, milk, fruits, and vegetables (Acharya and Prapulla 2010). XOS consist of xylose molecules connected by β (1–4) linkages with DP ranging from 2 to 10. Xylan, being the main hemicellulosic component in the lignocellulosic materials (LCMs), represents a potential source for XOS production (Jain et al., 2015). XOS can be produced by the hydrolysis of LCMs *via* chemical, enzymatic, or combination of chemical and enzymatic (chemo-enzymatic) methods at an industrial scale (Jain et al., 2015). The enzymatic process for XOS production from xylan has proven to be favorable for the manufacture of pharmaceutically important and food-grade XOS using food-grade xylanolytic enzymes (endo-xylanase, exo-xylanase, β-xylosidase, and debranching enzymes) (Acharya and Prapulla, 2011; Mamo et al., 2013). Attempts have also been made to use immobilized xylanase to produce XOS with lower DP (2 and 3) for its potential biotechnological applications (Sukri and Sakinah, 2018). Isomalto-oligosaccharides (IMO): IMO are made up of glucose units linked through α (1–6) glycosidic bonds using glucose units. Natural sources of IMO include honey, miso, soy sauce, and sake. Commercially, it is produced through a two-stage process using starch as a substrate. Starch is first hydrolyzed to liquefied starch by using a mixture of α-amylase and pullulanase. Then, α-amylase hydrolyses liquefied starch to maltose. In the second stage using the transglucosidase activity of α-glucosidase, IMO are produced from maltose (Callaway and Ricke, 2011). Other emerging prebiotic oligosaccharides: In addition to the above-mentioned well-researched NDOs, there are other oligosaccharides too which are emerging and have good potential. These include pectic-oligosaccharides (POS), manno-oligosaccharides (MO), and chitin-oligosaccharides (COS). POS are synthesized from pectin using pectinase. Many investigations have been directed at the use of pectin-rich agro-industrial by-products (citrus peel, orange peel, apple pomace) as a source of POS (Babbar et al., 2016). MO are produced by enzymatic hydrolysis of the commercial substrate using β-mannanase (Singh et al., 2018). Attempts have also been made to produce MO from mannan-rich agricultural by-products (Jian et al., 2013; Jana and Kango, 2020). COS can be isolated from natural sources or can be produced by chitin hydrolysis using chitinase (Ahmed et al., 2012; Qin and Zhao, 2019). Recently, the neuroprotective properties of feruloylated oligosaccharides (FOs) from maize bran have been evaluated (Li et al., 2020).

### Ideal Physicochemical and Biological Environment for Optimum Benefits of Prebiotics

Prebiotics promote the activation of microflora in the intestine and enhance the absorption of essential bioactive molecules through fermentation or other enzymatic activity. Therefore, the micro-environment in the gut plays a vital role in the optimum benefits of these compounds. The majority of the prebiotics are active at a pH range of 7.0–8.0. Other factors including temperature, oxygen status, digestive enzymes, and the presence of H_2_O_2_ are also known to influence the activity of both prebiotics and probiotics. Since fermentation is the major process in the enhancement of the growth of probiotics by prebiotics, the presence of antimicrobial peptides, immunoglobulin A, microRNA, and fecal microbiota transplantation are some of the non-specific host factors known to influence the outcome (Hasan and Yang 2019) and the host-associated factors are age, disease status, genetic makeup, diet, and lifestyle. The presence of metal ions is also known to influence the activity of prebiotics. Metal ions like Mg^2+^, Fe^2+,^ Mn^2+^, and Zn^2+^mediate enzyme activation and protein translation. Fe^2+^ and Mn^2+^ are said to be associated extensively with the ribosome and can replace the activity of Mg^2+^ (Bray et al., 2018). Therefore, it is essential to consider host, external, and other factors (diet, lifestyle use of drugs and antibiotics) while considering the health benefits or influence of prebiotics on metabolic changes in probiotics.

## Methodology

Biomedical literature databases (PubMed and Google Scholar) were searched using neuroprotection, oligosaccharides, prebiotics, non-digestible oligosaccharides, brain health, oxidative stress, or neurodegeneration as keywords within the article title. Peer-reviewed articles dealing with neuroprotective prebiotic oligosaccharides were critically read and included in the review. Research and review articles about preclinical or clinical trials on the therapeutic potential of prebiotics in brain health were analyzed for inclusion in this review. ClinicalTrails.gov website was referred to analyze the articles on human trials. At the preclinical and clinical level, analysis focused on prebiotics type and dose, subjects, mode of administration, treatment duration, and measurable biochemical outcomes.

## Therapeutic Spectrum of Prebiotic Oligosaccharides and Their Neuroprotective Effect: Validated Experimental Evidence on Probable Mechanisms

### Evidence in Animal Models

A substantial body of evidence suggests that SCFA produced *via* the fermentation of prebiotic NDOs by colonic probiotics plays a key role in maintaining brain homeostasis (van de Wouw et al., 2018; Silva et al., 2020). Results have indicated that approximately 500–600 nmol of SCFAs/day was produced in the molar proportion of 60:20:20 of acetate, propionate, and butyrate, respectively (Dalile et al., 2019). After their production in the colon, SCFAs subsequently get rapidly absorbed by colonocytes and the unabsorbed SCFAs get transported into the portal circulation. Furthermore, a small amount of the colon-derived SCFAs reaches the peripheral tissues and systematic circulation (Boets et al., 2015). The complete mechanism of how SCFAs influence brain function is not understood well.

Several animal studies have determined that SCFAs exert prevalent effects on key neurological and behavioral processes and are implicated in the crucial phases of neurodevelopmental and neurodegenerative disorders (Dalile et al., 2019; Fung et al., 2017) (Table 2). SCFAs influence brain functions by binding as endogenous ligands to G protein-coupled free fatty acid receptors (FFARs) and/or by inhibiting histone deacetylase in the brain (Vijay and Morris, 2014; Dalile et al., 2019). Multiple evidence has revealed that the functional SCFA receptors are localized in the gastrointestinal mucosa and the CNS (Parada Venegas et al., 2019). SCFAs are known to regulate systemic function through the inhibition of histone deacetylase activity (Lin et al., 2015). Histone deacetylase (HDAC) enzymes catalyze the exclusion of acetyl groups resulting in the interaction of the positively charged histones with the negatively charged DNA, thereby leading to a transcriptionally repressive, more compacted chromatin conformation (Licciardi et al., 2011). Intracellular butyrate and other SCFAs are reported to inhibit HDAC activity (Dalile et al., 2019), and the intracellular HDAC inhibition signaling found in the gut and remote organs is on binding to cell surface receptors (Stilling et al., 2016). However, the mechanism of this signaling is not clearly understood.

**TABLE 2:** Selected *in vivo* studies at a preclinical level using prebiotics and/or their metabolites (SCFA) and probiotics to modulate brain physiology and function.

Prebiotics/Probiotics/SCFA	Feeding duration	Model organism	Observation	References
Sodium butyrate (1.2 g/kg BW) in combination with fluoxetine (10 mg/kg BW)	4 weeks	C57BL/6J mice	Histone hyperacetylation in the hippocampus and frontal cortex for short-term, thereby exerted anti-depressant like effects	Schroeder et al. (2007)
Sodium butyrate (1.2 g/kg BW)	4 weeks	CK-p25 Tg mice	Histone hyperacetylation improved learning and memory	Fischer et al. (2007)
Propionic acid (500 mg/kg BW, twice a day)	3 weeks	Pregnant Long-Evans rats	Enhancement in repetitive behavior associated with ASD	Foley et al. (2014)
Sodium butyrate (1.2 g/kg BW)	5 weeks	Female ICR (CD1) mice	Increased histone H4 acetylation in hippocampus facilitated amelioration of memory impairment	Takuma et al. (2014)
GOS (4 g/kg BW)	Postnatal days (3–21)	Neonatal rats	Alterations in BDNF levels, synaptic proteins (synaptophysin, MAP2, and GAP43), and NMDAR subunits (GluN1, GluN2A, GluN2B)	Williams et al. (2016)
*E. faecium* (4 × 10^8^ CFU)	5 weeks	Sprague-Dawley male rats	Lower levels of pro-inflammatory cytokines and higher levels of BDNF in the hippocampus region of synbiotic and prebiotic treated animals Significant increase in butyrate concentration after synbiotic and prebiotic supplementation	Araiza et al., 2018
Inulin (860 mg/kg BW)				
*E. faecium* + inulin (4 × 10^8^ CFU + 860 mg/kg BW)				
GOS (15 g/L)	3 weeks	SPF Sprague-Dawley male adult rats	Downregulated activation of microglial cells, thereby reducing surgery-induced cognitive impairments	Yang et al. (2018)
Polydextrose-GOS (7 and 15 g/kg BW for mice and rats, respectively)	Postnatal days (21–50)	Weanling male C57BL/6J mice SD rats LE rats	Improvement of memory and reducing anxiety-related behaviors in normally developing rodents	Waworuntu et al. (2014)
Polydextrose-GOS (2 g/L each)	Postnatal days (2–33)	Translational piglet model	Higher recognition memory	Fleming et al. (2019)
FOS GOS FOS + GOS (0.3–0.4 g/mouse/day)	10 weeks	Male C57BL/6J mice	Improvement in brain chemistry and social behavior related to anxiety and depression Enhanced levels of SCFA in the caecum reduction in stress-induced corticosterone levels in plasma Anti-anxiety levels in open field and elevated plus-maze	Burokas et al. (2017)
XOS (10%)	12 weeks	Male Wister rats	Enhancement in brain mitochondrial function and synaptic plasticity, thereby restoring cognitive function	Chunchai et al. (2018)
*L. paracasei* HIIO1 (1 × 10^8^ CFU)				
Synbiotics (XOS + *L. paracasei* HIIO1; 1:1 ratio)				
Lactoferrin (0.3 g/100 g milk powder)	Postnatal days (2–31)	Piglets	Positively influenced brain development as evidenced with neuroimaging outcomes	Mudd et al. (2016)
Milk fat globule membrane (0.25 g/100 g milk powder)				
Blend of polydextrose (1.3 g/100 g milk powder)/GOS (3.5 g/100 g milk powder)				
FOS + XOS (3 g/kg/day)	Gestation days (0–19)	Pregnant Wistar rats	Enhanced exploratory behavior in the open field test Reduction in acrylamide-induced oxidative stress markers level	Krishna et al. (2015)
Inulin (2 g/kg/day)	Gestation days (0–19)	Pregnant Wistar rats	Reduction in acrylamide induced increase in oxidative markers in the fetal and the brain tissues	Krishna and Muralidhara (2015)
Inulin (2 g/kg BW, twice a day)	Gestation days (6–19)	Pregnant Wistar rats	Inulin supplementation diminished gestational rotenone induced increase in oxidative markers in the regions of the maternal brain and affected the whole fetal brain	Krishna and Muralidhara (2018)
FOs (200 ul)	4 weeks	Young adult female C57/BL mice	Ameliorated behavioral recovery following spinal cord injury *via* modulating the expression of inflammatory mediators	Li et al. (2020)
COS (0.2 mM)	1 day	*C. elegans*	Enhancement in the antioxidant potential with an increase in dopamine levels, thereby attenuating monocrotophos induced oxidative stress	Nidheesh et al. (2016)

Psychiatric disorders including depression and anxiety are closely associated with histone acetylation (Stilling et al., 2016). In animal models, SCFA-induced histone hyperacetylation such as a reduction in depressive behavior has been observed (Schroeder et al., 2007; Wei et al., 2015). Schroeder et al. (2007) demonstrated the ability of sodium butyrate (single dose: 1.2 g/kg BW) as an HDAC inhibitor in combination with a selective serotonin reuptake inhibitor, fluoxetine (10 mg/kg BW, antidepressant drug). Acute and chronic administration of sodium butyrate alone or in combination with fluoxetine in mice (C57BL/6J) for a period of 28 days induced short-lasting histone hyperacetylation in the hippocampus and frontal cortex, thereby exerting anti-depressant like effects. On similar lines, histone hyperacetylation following intraperitoneal injection of sodium butyrate (1.2 g/kg BW daily for 4 weeks) in CK-p25 Tg mice improved learning and memory in wild-type mice and in mice with brain atrophy (Fischer et al., 2007). Conversely, excessive levels of SCFA might have had adverse effects on brain health and behavior. Elevations in propionic acid are shown to induce autism-like symptoms in animal models through the formation of propionyl coenzyme A (CoA) and sequestration of carnitine (MacFabe, 2012; MacFabe, 2015). In another study, prenatal and early life administration of propionic acid (500 mg/kg BW, twice a day for 3 weeks) to pregnant Long-Evans rats on gestation days G12–16 resulted in enhanced repetitive behavior in the open-field test contributing to ASD (Foley et al., 2014). Prenatal exposure to valproic acid results in autism spectrum disorder (ASD) with a spatial learning disability and anxiety-like behavior. Interestingly, chronic treatment (5 weeks) to female ICR (CD1) mice with sodium butyrate (1.2 g/kg BW/day, i.p.; starting at 4 weeks of age) reversed valproic acid-induced ASD-like behavior in the offspring. Increased histone H4 acetylation with sodium butyrate administration in the mice hippocampus facilitated the amelioration of the memory impairment in prenatally valproic acid-exposed mice (Takuma et al., 2014). Therefore, these research studies suggest that extreme care should be taken to evaluate the potential use of SCFAs to treat ASD (Stilling et al., 2016).

Aging is associated with degenerative loss of neurons in the CNS, ultimately leading to memory loss and impaired learning (Romo-Araiza et al., 2018). Furthermore, it is also associated with the reduced expression of brain-derived neurotrophic factors (BDNFs) in the hippocampal region that is closely associated with the regulation of synaptic transmission and plasticity (Ryan and Nolan, 2016). Thus, aging is associated with reducing levels of BDNF. The maintenance of sufficient BDNF concentrations is suggested to delay the onset of cognitive impairments (Pineda-Rodriguez et al., 2017). Several studies have highlighted the role of prebiotics and their metabolites (SCFA) in psychophysiological modulation *via* BDNF (Sarkar et al., 2016; Park et al., 2017; Haghighat et al., 2019; Heyck and Ibarra, 2019). Williams et al. (2016) studied the effect of GOS supplementation on brain development and maturation in neonatal rats from the postnatal days (3–21). GOS supplementation brought alterations in the levels of BDNF, synaptic proteins (synaptophysin, MAP2, and GAP43) and NMDAR subunits (GluN1, GluN2A, GluN2B) on postnatal days (22 and 56). An increase in the levels of BDNF, NMDAR subunit, GluN2A, and synaptic protein, *viz*., synaptophysin (but not MAP2) imply that neonatal GOS supplementation alters neurotransmission instead of synaptic architecture. Thus, the study confirms that prebiotic oligosaccharides are capable of manipulating gut microbiota in early life and its positive effects on the brain persist at least up to young adulthood. On similar lines, Romo-Araiza et al. (2018) investigated the modification of intestinal microbiota through supplementation of prebiotics/probiotics/synbiotics and thereby studied their effect on brain health. Middle-aged Sprague-Dawley male rats were randomly assigned into 4 groups and each group (one group served as a control) was supplemented (5 weeks through oral gavaging) with either probiotic *Enterococcus faecium* (4 × 10^8^ CFU) or prebiotic, inulin (860 mg/kg BW) or synbiotic, comprising of *E. faecium* and inulin (4 × 10^8^ CFU + 860 mg/kg BW). The impact of probiotic, prebiotic, or synbiotic supplementation on spatial and associative memory in middle-aged rats was assessed at the end of the study. Their findings revealed that synbiotic and prebiotic supplemented groups performed significantly better in the spatial memory test. This improvement is correlated with lower levels of pro-inflammatory cytokines and higher levels of BDNF in the hippocampus region of synbiotic and prebiotic-treated animals. A significant increase in butyrate concentration after synbiotic and prebiotic supplementation is potentially the reason for the enhanced levels of BDNF and progression of spatial memory. Delta butyrate concentration for each group was found as follows: control (0.45 ± 0.008), probiotic (−0.008 ± 0.009), prebiotic (0.87 ± 0.008), and synbiotic (1.17 ± 0.01). These values clearly indicated that the inulin supplementation resulted in the improvement of butyrate concentrations. Furthermore, a decrease in pro-inflammatory cytokine concentrations and an increase in BDNF levels in the hippocampus region offered a positive outcome. Thus, these research findings clearly indicate that supplementation of prebiotics alone or in combination with probiotics positively impacts brain health *via* modulation of BDNF.

Microglia are reported to play a crucial role in brain development and therefore represent the principal immune cells in the human brain (Chen and Trapp, 2016). These cells populate the CNS *in utero* and assist in brain development. Once neuronal development is complete, they serve as resident innate immune cells of the CNS and get activated only when the CNS is challenged with injury, infection, and/or disease. During the inflammatory state, microglial activation is not only associated with neuroprotective effects (*viz.*, phagocytosis of dead neurons and clearance of debris) but also has neurotoxic consequences (Polazzi and Monti, 2010). Thus, microglia are the first cells to induce neuroinflammatory response and thereby play a crucial role in the initiation of various mental disorders (Yang et al., 2018). It is found that postoperative cognitive dysfunction, Alzheimer’s and Parkinson’s diseases are associated with enhanced concentrations of pro-inflammatory cytokines along with microglial activation in the brain (Chunchai et al., 2018; Yang et al., 2018; Liu et al., 2019). Recent studies have identified a communication link between prebiotic NDOs and microglia. Yang et al. (2018) evaluated whether supplementation of GOS would attenuate postoperative cognitive dysfunction and surgery-induced neuroinflammation. To assess the effect of GOS supplementation, abdominal surgery under isoflurane anesthesia was performed on SPF Sprague-Dawley male adult rats. Over the course of 3 weeks of the study, the GOS-treated group received GOS at a dose of 15 g/L in water. At the end of the study, supplementation of GOS significantly attenuated surgery-induced cognitive impairments and downregulated activation of microglial cells in comparison to the control group. A detailed analysis of the gut revealed that GOS supplementation significantly altered the diversity of the gut microbiome and enhanced the proliferation of anti-inflammatory microbes, *viz.*, bifidobacteria*.*

Dietary oligosaccharides have shown potential to improve memory, cognition ability, and social behavior (Waworuntu et al., 2014; Collins and Reid, 2016). A mixture of polydextrose-GOS fed to mice (15 g/kg BW) and rats (7 g/kg BW) from postnatal days 21–50 showed increased positive social interactions and higher object recognition index in comparison to the control group. Thus, these results indicate the ability of the tested prebiotics in the improvement of memory and reduced anxiety-related behavior in normally developing rodents (Waworuntu et al., 2014). The same mixture (2 g/L each) was further evaluated by Fleming et al. (2019) in young pigs administered from postnatal days 2–33. The study found that early life consumption of this mixture supported higher recognition memory. In line with this, the beneficial role of FOS and GOS in stress-related behavior has been evaluated (Burokas et al., 2017). Male C57BL/6J mice that received either FOS, GOS, or both at a dose of 0.3–0.4 g/mouse/day for 10 weeks showed improvement in brain chemistry and social behavior related to anxiety and depression. Moreover, combination treatment was found to be more effective in altering the microbial community, and this led to the increase in SCFA levels in the caecum. Furthermore, marked reduction in stress-induced corticosterone levels in plasma and anti-anxiety levels in an open field and elevated plus-maze were recorded. All these evidences confirm the potential of combination treatment, that is, polydextrose-GOS and FOS-GOS in the successful reduction of anxiety-related symptoms.

High-fat diets can cause cognitive decline and microglial hyperactivity in addition to obesity-induced insulin resistance. In line with this, Chunchai et al. (2018) explored the effect of prebiotics (XOS; 10%), probiotics (*L. paracasei* HIIO1; 1 × 10^8^ CFU), and synbiotics (XOS + *L. paracasei* HIIO1; 1:1 ratio) on microglia in obese-insulin-resistant male Wistar rats. Male Wister rats used in the study for 12 weeks were fed with either a high-fat or normal diet. At the beginning of the 13th week, the rats were randomly assigned into 4 subgroups in each of the dietary treatments (vehicle, prebiotic, probiotic, and synbiotic groups) followed by intervention with the assigned dietary supplement. After 12 weeks of treatment, the cognitive function and microglial activation status of each rat group were assessed. Prebiotic, probiotic, and symbiotic-fed groups showed a significant decrease in high-fat diet-induced cognitive impairments and microglia activation. Prebiotic, probiotic, and synbiotic treatments could significantly reduce microglial activation. Overall, the animals that received the supplements had better cognitive functions in comparison to the control group clearly suggesting that prebiotic, probiotic, and synbiotic treatments could effectively attenuate cognitive impairments and inhibit microglial activation thereby conferring neuroprotection.

Like all other tissues in the human body, brain tissues require oxygen to meet their energy needs. The human brain constitutes 2% of total body mass and needs 20% of total body oxygen demand to support neuronal activity (Devi and Satpati, 2017). The oxygen consumption rate of the normal human brain is 3.5 ml/min/100 g brain tissue (Rink and Khanna, 2011). The brain is an active site for the production of free radicals from reactive oxygen species (ROS) as a result of inefficiencies in oxidative phosphorylation in the mitochondria (Black et al., 2015). An excessive level of free radicals can impair brain function. Therefore, a balance between free radicals and antioxidants is essential to support brain function from any degeneration. Several defensive mechanisms exist to counteract and protect brain cells against oxidative stress-mediated neuronal degeneration such as upregulation of endogenous antioxidants and removal of damaged proteins and organelles by autophagy (Divyashri et al., 2015). However, higher rates of oxidative stress can initiate the oxidation of proteins and lipids, thereby changing their structure and functions that can subsequently result in cell death (Dumitrescu et al., 2018; Lobo et al., 2010). Therefore, any excess of free radical production due to oxidative stress could be associated with damage to a wide range of molecular species. Studies on prebiotic NDOs alone or in combination with probiotics have the potential to modulate and counter oxidative stress in the brain. This is gaining significant attention and relevance as a strategy to treat/prevent neurological pathologies, including Alzheimer’s and Parkinson’s diseases.

Early life intake of key nutritional components (prebiotics) enriches neurodevelopmental activities (Kao et al., 2016). The beneficial effects of lactoferrin (0.3 g/100 g milk powder), milk fat globule membrane (0.25 g/100 g milk powder), and a blend of polydextrose (1.3 g/100 g milk powder)/GOS (3.5 g/100 g milk powder) as potent prebiotics on the early developing brain are elucidated using piglets (dosage from 2–31 days). It is notable that dietary supplementation was well tolerated and positively influenced brain development (Mudd et al., 2016). Krishna et al. (2015) evaluated the physiological benefits of prebiotic oligosaccharides during pregnancy. Attempts were made to test the effectiveness of a prebiotic combination (FOS + XOS) to attenuate acrylamide-induced oxidative impairments, mitochondrial dysfunction, and neurotoxicity in maternal and fetal rat brains. A dose of 3 g/kg/day of oligosaccharides (XOS and FOS) was supplemented to pregnant dams (Wistar rats) during the gestation period of 0–19 days. Simultaneously, the rats were exposed to acrylamide (200 ppm in drinking water). Prebiotics fed acrylamide dams displayed better exploratory behavior in the open field test. Furthermore, prenatal assessment proved that prebiotic supplementation could effectively restore acrylamide-induced decrements of placental/fetal weights. In addition, prebiotics supplementation could significantly lower the markers of oxidative stress (ROS, reduced glutathione, and protein carbonyls) and restore activities of antioxidant enzymes (acetylcholinesterase and glutathione peroxidase), in the maternal and fetal brains with a concomitant increase in dopamine and γ-aminobutyric acid levels. This study suggested that prenatal prebiotic oligosaccharide supplements can effectively safeguard the developing brain against acrylamide-induced oxidative stress-mediated neurotoxicity.

Scientific evidence suggests that the consumption of prebiotic oligosaccharides influences the human brain positively. The influence of inulin supplementation during gestation in acrylamide-induced oxidative impairments and neurotoxicity in maternal and fetal rat brains was examined by Krishna and Muralidhara (2015). Pregnant rats were co-fed with inulin (2 g/kg/day; gestation days 0–19) and acrylamide at a dose of 0.2 g/L (gestation days 6–19). Their results revealed that inulin supplementation could significantly increase placental weight among acrylamide-exposed rats. A detailed analysis of oxidative stress markers (ROS, hydroperoxide, lipid peroxidation, reduced glutathione, and protein carbonyls) revealed that inulin supplementation could effectively lower acrylamide-induced increase in oxidative markers in the fetal and the brain tissues. This study reveals the neuroprotective role of prebiotic NDOs towards developmental neurotoxicants such as acrylamide during pregnancy.

The impact of increased pesticide exposure on developmental neurotoxicity due to entry into the immature brain is of concern. Investigations have identified the influence of maternal gut microbiota on utero fetal development by modulating the host gut microbial composition with prebiotic NDOs. Krishna and Muralidhara (2018) examined the beneficial role of inulin in a developmental model of rotenone neurotoxicity. Pregnant rats gavaged orally with inulin (2×/day, 2 g/kg BW; during gestation days 0–21), also received rotenone (50 mg/kg BW, gestation days 6–19) to potentially counter the developmental effects of general fetotoxicity, cholinergic activities, and oxidative stress in maternal and whole fetal brain. It was found that inulin supplementation resulted in a significant increase in maternal caecal bacterial numbers, with a concomitant increase in exploratory behavior among rotenone-treated rats. Furthermore, inulin supplementation diminished gestational rotenone-induced increase in oxidative markers (ROS protein carbonyls and lipid peroxidation) in the regions of the maternal brain (cerebellum, striatum, and cortex), and the fetal brain. This study indicates the prebiotics potential in lowering oxidative stress-mediated neurodegenerative disorders.

Deterioration in neuronal survival and irreversible motor and sensory dysfunction is reported to occur following spinal cord injury. The neuroprotective effect of FOs (200 ul, 4 weeks) was evaluated using the spinal cord injury model of young adult female C57/BL mice. FOs modulated the expression of inflammatory mediators (downregulated TNF-α, IL-2, IL-6, IL-18 levels, and upregulated IL-10 and BDNF) and ameliorated behavioral recovery following spinal cord injury. Furthermore, the study demonstrated that FOs exhibited anti-inflammatory and neuroprotective effects *via* the mitogen-activated protein kinase signaling pathway with enhanced expression of BDNF levels (Li et al., 2020).

Many studies have attempted to use models outside of the rodent system to evaluate the neuroprotective potential. *Drosophila melanogaster* is a small insect, encompasses noteworthy cellular, molecular, and biological signaling complexity with relevance to humans (Westfall et al., 2018). Thus, *Drosophila* has become one of the prominent model systems to evaluate neuroprotection. However, investigations on the effect of NDOs on neurological disorders using *Drosophila* are limited. *Caenorhabditis elegans* also represents a model system for studying neuroprotective effects as it has relevant homology with mammalian systems. However, investigations on the NDOs effect on neurological disorders using *C. elegans* are comparatively few. A study by Nidheesh et al. (2016) tested the efficiency of chito-oligomers (COS) to ameliorate monocrotophos-induced oxidative stress in *C. elegans*. COS exhibited a significant neuroprotective effect by enhancing the antioxidant potential of the brain and thereby attenuating oxidative stress. But this systematic review provides an interesting perspective on the current evidence base for the effects of prebiotics on symptoms of oxidative stress reduction and improving brain health using rodent models.

### Evidence in Humans

Prebiotic administration at the clinical level to investigate their central effects as neuroprotective agents is currently lacking (Kao et al., 2016; Johnstone et al., 2021). However, we have summarized the limited progress made in the clinical studies with NDOs as medicinal therapeutics (Table 3). A study in 45 healthy male volunteers found that the consumption of FOS (5.5 g/day) and GOS (5.5 g/day) for 2 weeks helped to proliferate host gut microbiota with a reduction in salivary cortisol levels. In comparison to FOS, GOS were more successful in regulating the hypothalamic–pituitary–adrenal (HPA) axis to restore emotional perturbations in healthy volunteers (Schmidt et al., 2015). This study demonstrates the ability of prebiotic oligosaccharides in the reduction of anxiety-related psychological mechanisms. A randomized double-blind study to assess the effect of short-chain FOS (5 g/day; 4 weeks) on clinical outcomes of anxiety/depression was performed on 79 irritable bowel syndrome (IBS) volunteers. FOS significantly reduced anxiety by modulating gut microbiota (Azpiroz et al., 2017). The influence of GOS (7.5 g/day for 4 weeks) on gut microbiota to improve mental ailments, *viz.*, anxiety and moodiness was evaluated in late adolescence and early adulthood using healthy female volunteers (64 no.; aged between 18–25 years) (Johnstone et al., 2021). The authors reported that through a dot-probe task, GOS reduced negative emotional bias and increased positive bias associated with high anxious participants. This finding was further supported by the increased beneficial bacterial abundance in the GOS supplemented group.

**TABLE 3:** Selected studies at a clinical level using prebiotics and/or their metabolites (SCFA) and probiotics to modulate brain physiology and function.

Prebiotics and/or Probiotics	Duration	Subjects	Outcome	References
FOS (5.5 g/day)	2 weeks	45 healthy male and female volunteers	Reduction in salivary cortisol levels Regulation of HPA axis to restore emotional perturbations	Schmidt et al. (2015)
GOS (5.5 g/day)				
FOS (5 g/day)	4 weeks	79 irritable bowel syndrome (IBS) volunteers	Reduction in anxiety by modulating gut microbiota	Azpiroz et al. (2017)
GOS (7.5 g/day)	4 weeks	64 healthy female volunteers	Increased beneficial bacterial abundances Reduction in negative emotional bias and increase in positive bias associated with high anxious participants	Johnstone et al. (2021)
Mixture of GOS/FOS/pectic-derived acidic oligosaccharides (1.5 g/kg BW/day)	Gestation days (3–30)	77 preterm infants with a gestational age of less than 32 weeks	No improvement in the neurodevelopmental outcome was observed at the corrected age of 2 years	van den Berg et al. (2016)
Mixture of GOS/FOS and pectic-derived acidic oligosaccharides (80% + 20%)	Gestation days (3–30)	93 preterm infants with a gestational age of less than 32 weeks	No beneficial effect on neurodevelopmental outcome in preterm infants in the first year of life	LeCouffe et al. (2014)
Oligofructose-enriched inulin (5 g/day)	1 week	47 subjects	Selective improvement was observed with recall and recognition memory No effect on mood and sustained behavior was reported	Smith et al. (2015)
Probiotic mixture (5 g) of 2.7 × 10^7^ CFU/g each of *L. acidophilus* T16, *B. bifidum* BIA-6, *B. lactis* BIA-7, and *B. longum* BIA-8	12 weeks	75 hemodialysis patients	Reduction in depression associated symptoms with a concomitant increase in serum BDNF levels in the synbiotic treated group	Haghighat et al. (2019)
Prebiotic mixture: 5 g each of FOS, GOS, and inulin				
Synbiotic mixture: 15 g prebiotics and 5 g probiotics				

It is proven at the preclinical level that the early life intake of various prebiotic oligosaccharides supported neurodevelopmental activities (Krishna et al., 2015; Fleming et al., 2019; Upadhyay et al., 2020). However, at the clinical level, these studies were examined with a smaller experimental window. Preterm infants (77 no.) with a gestational age of <32 underwent a randomized double-blind trial and received a mixture of short-chain GOS/long-chain FOS/pectic-derived acidic oligosaccharides (1.5 g/kg BW/day) through breast/formula milk from day 3 to 30 (van den Berg et al., 2016). Neurodevelopmental outcome at the corrected age of 2 years revealed that the supplementation of the above mixture resulted in no improvement in comparison to the placebo group. This is attributed to the higher serum cytokine levels with lower bifidobacterial counts indicating the importance of gut microbiota in the immune response during the brain development process. A similar observation was made by LeCouffe et al. (2014) wherein enteral supplementation of a prebiotic mixture (for the short term) displayed no beneficial effect on neurodevelopmental outcome in preterm infants in the first year of life. Although these studies are acknowledged for conducting prebiotic research involving preterm infants, the results seem not conclusive even though the beneficial effects of early life intake of prebiotic oligosaccharides in rodent models are well-established. Therefore, further studies are required to evaluate the early life intake of prebiotic oligosaccharides on neurodevelopment to draw accurate conclusions. The effect of a prebiotic intervention on human mood, learning, affective, and cognitive processes was previously reported. Smith et al. (2015) involved 47 subjects (1 week) to evaluate the effect of oligofructose-enriched inulin (5 g/day) on human well-being and cognitive performance. Selective improvement following inulin ingestion was observed with recall and recognition memory. However, no effect on mood and sustained behavior was observed.

Hemodialysis procedure is reported to have an adverse effect on the patient’s mental health (Teles et al., 2014). Researchers in this study were also making efforts to combine prebiotics with specific probiotics for the formulation of a symbiotic mixture to maximize their health benefits. To investigate the beneficial effect of prebiotics/probiotics/synbiotics in hemodialysis patients suffering from anxiety and depression, Haghighat et al. (2019) enrolled 75 patients for the study. The patients (*n* = 75) were randomly assigned to synbiotic group (*n* = 25; prebiotics, 15 g and probiotic mixture, 5 g), probiotic group (*n* = 25; probiotic mixture, 5 g and maltodextrin, 15 g), and a placebo group (*n* = 25; maltodextrin, 20 g). Probiotic mixture (5 g) comprised of 2.7 × 10^7^ CFU/g each of *Lactobacillus acidophilus* T16, *Bifidobacterium bifidum* BIA-6, *Bifidobacterium lactis* BIA-7, and *Bifidobacterium longum* BIA-8. The prebiotic group received 5 g each of FOS, GOS, and inulin. Synbiotic supplementation for 12 weeks resulted in a greater decrease in depression symptoms measured as Hospital Anxiety and Depression Scale (HADS) (HADS-DEP ≥ 8) with a concomitant increase in serum BDNF in comparison to the probiotics and placebo groups.

### Mechanism-Based Studies

**FIGURE 2:**
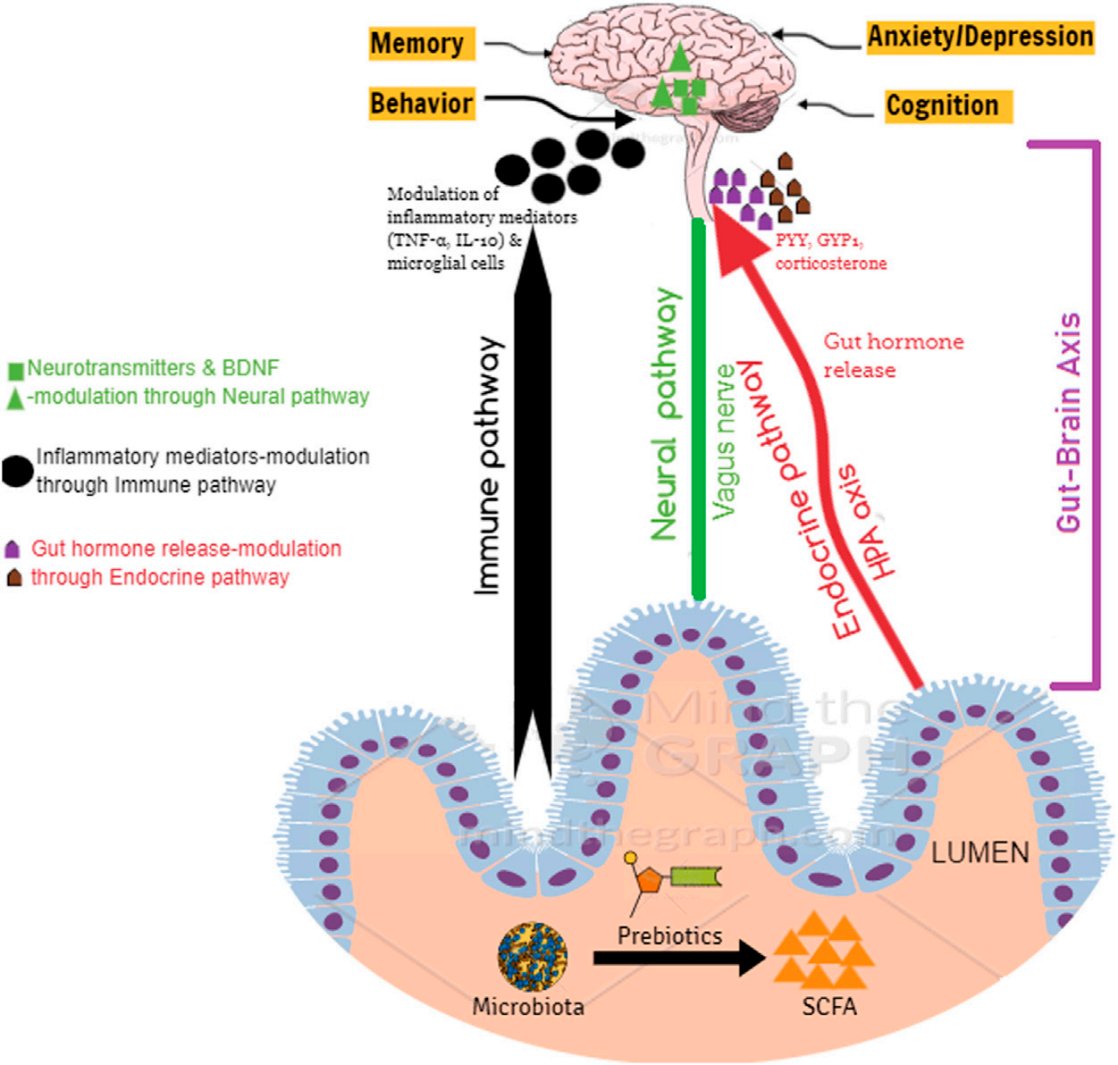
Protective mechanism of prebiotic NDOs on the brain.

SCFAs provide benefits to peripheral tissues, and therefore, the colon is well supported, and it is also suggested that potentially they exert crucial physiological effects on distal organs, including the brain (Silva et al., 2020). SCFAs can cross the blood–brain barrier *via* monocarboxylate transporters located on endothelial cells, and hence, it can alter the neurotransmitter (γ-aminobutyric acid (GABA), and serotonin (5-HT)) and hormone concentrations (glucagon-like peptide 1 (GLP1) and peptide YY (PYY)) by promoting their secretion (Silva et al., 2020). In addition, they are also reported to prevent neurodegeneration and promote neuronal regeneration (Sampson and Mazmanian, 2015) and thereby have a protective effect on the brain *via* direct and indirect means (Figure 2). Direct pathways influencing brain function: SCFAs can cross the blood–brain barrier to reach the brain, and the average concentrations of butyrate and propionate in the human brain tissue were found to be 17.0 and 18.8 pmol/mg of brain tissue, respectively (Silva et al., 2020). Further evidence suggests that SCFAs can exhibit neuroactive properties in the CNS (Tran and Mohajeri, 2021). However, the mechanism by which these SCFAs offer neuroprotection is still not clear. Indirect pathways influencing brain function: Gutbrain axis is composed of the CNS, enteric nervous system (ENS), afferent, and efferent neurons that are associated with the signal transduction between the gut and the brain (Chen et al., 2017; Liu et al., 2018). Numerous scientific evidences indicate the widespread communication between the gut and the brain *via* the gutbrain axis (Chen et al., 2013; Liu et al., 2015). The bidirectional communication between the gut and the brain occurs through the vagus nerve, neuroimmune, humoral, and neuroendocrine pathways (Sandhu et al., 2017). By interacting with FFAR receptors on enteroendocrine cells, SCFA promotes indirect signaling to the brain *via* the systemic circulation or vagal pathways by inducing the secretion of neurotransmitters (GABA and 5-HT) and gut hormones (GLP1 and PYY) (Silva et al., 2020). SCFAs, particularly butyrate is known to influence brain activity indirectly by acting through the gutbrain axis (Stilling et al., 2016). Butyrate, with its ability to cross the blood–brain barrier, is reported to activate the vagus nerve, thereby indirectly influencing the brain by modulating host appetite and eating behavior (van de Wouw et al., 2017). Furthermore, butyrate also modulates the activity of cholinergic enteric neurons through epigenetic mechanisms (Soret et al., 2010). Butyrate, upon binding to its specific receptors in the intestine, is recognized to modulate signals to the brain *via* the gutbrain neural circuit through cAMP signaling pathways (Keenan et al., 2015).

Surgical trauma is reported to attenuate behavioral deficits, thereby enhancing neuroinflammatory responses with reduced SCFA and BDNF levels. The pretreatment effect (4 weeks) of exogenous SCFA to restore physiological and behavioral deficits was studied using 8- to 10-week-old adult male C57BL/6J mice (Xu et al., 2021). Mice were randomly assigned into 5 groups. First group served as control; second group of mice received SCFA (67. 5 mM sodium acetate, 25 mM sodium propionate, 40 mM sodium butyrate); third group served as surgery control; fourth group underwent surgery and received SCFA (dose similar to the second group); and fifth group received fecal microbiota transplantation. The impact of SCFA supplementation prior to surgery on spatial learning and memory was assessed at the end of the study. Their findings revealed that pretreatment with SCFA prior to surgery partially improved the locomotor activity and anxiety-like behaviors. SCFA feeding has also been shown to result in the reduction of surgical trauma-induced upregulation of IL-1β and IL-6 in the hippocampus region. Furthermore, upregulated levels of hippocampal Iba-1 levels were downregulated, thereby suggesting that SCFA treatment could effectively reverse microglial overactivation. This data clearly demonstrates the ability of exogenously administrated SCFA or gut-derived SCFA in the restoration of microglial over-activation and modulation of neuroinflammatory responses to rescue surgical trauma.

The neuroprotective effect of exogenously administered sodium butyrate was explored by Sun et al. (2016) using chronic unpredictable mild stress (CUMS)-induced male C57BL/B6 mice model. Sodium butyrate treatment (200 mg/kg BW, 2 weeks) reversed CUMS induced depressive status by enhancing BDNF expression with a concomitant increase in 5-HT concentration in the hippocampus region of mice brain. These data clearly suggest the ability of SCFA, more specifically butyrate in amelioration of depressive behaviors by enhancing 5-HT and BDNF levels.

Intragastric injections of sodium butyrate for 2 weeks on chronic unpredictable mild stress (CUMS)-induced depression-like behaviors in male C57BL/B6 mice was studied (Sun et al., 2016). It was observed that butyrate could ameliorate CUMS-induced alterations in BDNF expressions, thereby offering anti-depressive effects. Another validated mechanism by which SCFA offers neuroprotection is by modulation of the gut hormone release from enteroendocrine cells (Kao et al., 2016). SCFA produced through the ingestion of prebiotic oligosaccharides modulates the secretion of peptide tyrosine (PYY) and glucagon-like peptide-1 (GLP-1) from enteric L-cells. The effect of intra-colonic administration of propionic acid (180 mmol/L) on the release of GLP-1 and PYY was investigated using rodent models (male C57BL6 Wistar rats). Analysis of plasma gut hormone profile using radio-immunoassay revealed that the propionic acid administration stimulated the release of GLP-1 and PYY, thereby playing a significant role in the central effects (Psichas et al., 2015). It is a well-established fact in animal models that exogenous PYY influences behavioral and cognitive functions (Stadlbauer et al., 2015). Furthermore, PYY is also known to modulate vagal nerve activity through local BNDF signaling pathways (Kao et al., 2015). In addition to the above studies on PYY secretion and modulation by SCFA, prebiotic oligosaccharide, *viz.*, GOS (6%; 3 weeks) are also known to induce the expression of circulating PYY in male Wister rats (Overduin et al., 2013). GOS fed rats expressed elevated levels of PYY and GLP-P in colonic mucosal, thereby influencing brain health. These studies clearly explain the possible mechanisms by which NDOs and their products (SCFA) modulate brain chemistry.

In rodent models, HDAC inhibition with altered GABAergic signaling is closely connected to autism spectrum disorders (ASD) (Stilling et al., 2016). The effect of butyrate (i.p. 100 mg/kg BW/day, 10 days) on ASD-associated social behavior was evaluated by Kratsman et al. (2016) using the BTBR mouse model of autism. Administered dose of butyrate affected genes involved in neuronal excitation and inhibition. An increase in inhibitory neurotransmitter genes (Drd2 and Gabrg1) with the decrease in neuronal activation and excitatory neurotransmitter marker genes (cFos Grin2b, and Adra1) supports the fact that the butyrate promotes the transcription of inhibitory pathway transcripts. Furthermore, the tested dose of butyrate failed to induce any significant difference in histone acetylation in the prefrontal cortex; however, there was an increase in ASD-associated social behavior through the modulation of the excitatory/inhibitory balance.

All these evidences prove that prebiotics and their metabolites communicate to the brain *via* neural (vagus nerve, BDNF, neurotransmitters), endocrine (HPA axis and associated hormones), and immune (immune cells and markers, *viz*., TNF-α and IP-10) pathways (Table 4).

**TABLE 4:** Mechanism-based evidences of prebiotics and/or their metabolites (SCFA) to modulate brain physiology and function.

Pathway	Prebiotics and/or their metabolites (SCFA)	Model	Mechanism	Reference
Immune	SCFA mixture (67. 5 mM sodium acetate, 25 mM sodium propionate, 40 mM sodium butyrate)	Surgical trauma-induced adult male C57BL/6J mice model	Partial improvement in the locomotor activity and anxiety-like behaviors by The upregulation of IL-1β and IL-6 in the hippocampus region with the restoration of surgery-induced microglial over-activation.	Xu et al. (2021)
Neural	Sodium butyrate (200 mg/kg BW)	Chronic unpredictable mild stress (CUMS)-induced male C57BL/B6 mice model	Amelioration of CUMS induced alterations in BDNF expressions, thereby offering anti-depressive effects.	Sun et al. (2016)
Endocrine	Propionic acid (180 mmol/L)	Male C57BL6 Wistar rats	Modulates the secretion of gut hormones, PYY, and GLP-1 from enteric L-cells	Psichas et al. (2015)
Endocrine	GOS (6%)	Male Wistar rats	Elevations in the levels of PYY and GLP-P in colonic mucosal, thereby influencing brain health	Overduin et al., 2013
Neural	Sodium butyrate (100 mg/kg BW/day)	BTBR mouse model of autism	Promotes transcription of inhibitory pathway transcripts *via* an increase in inhibitory neurotransmitter genes (Drd2 and Gabrg1) with a decrease in neuronal activation and excitatory neurotransmitter marker genes (cFos Grin2b, and Adra1)	Kratsman et al., 2016
Immune	COS (200, 400 or 800 mg/kg BW)	Amyloid-β-_1–42_-induced Alzheimer’s disease (AD) rats	Reduction in the levels of IL-1β and TNF-α to influence cognitive functions	Jia et al. (2016)
Immune	FOS (2.5 and 5%)	D-Galactose AD rat model	Improvement in spatial learning and memory by reducing Aβ density in the cortex and hippocampus with improvement in the plasma ascorbic acid level in a dose-dependent manner	Yen et al. (2017)
Neural	FOS extract (50 and 100 mg/kg BW/day)	D-Galactose AD rat model	Enhancement in the levels of neurotransmitters (norepinephrine, dopamine, 5-hydroxytryptamine, and 5-hydroxyindole acetic acid) with down-regulate the expression of AD-related intracellular markers (Tau and Aβ_1−42_)	Chen et al. (2017)
Immune	Sodium butyrate (300 mg/kg BW)	Hypoxic–ischemic-injured immature rat model	Stimulation of oligodendrocyte precursor cell proliferation in the hippocampal dentate gyrus with a reduction in the microglial cell number in the rat ipsilateral hemisphere and enhancement in the BDNF levels	Ziemka-Nalecz et al. (2017)
Neural	Sodium butyrate (300 mg/kg BW)	Neonatal rat model of hypoxia–ischemia	Increased BDNF levels with enhanced activation of the TrkB receptor (BDNF receptor) and the phosphorylation of the transcription factor-CREB-in the ipsilateral hemisphere suggests the involvement of BDNF-TrkB signaling pathways	Jaworska et al. (2019)
Immune	COS (0.1 ml/10 g BW)	Neonatal rat model of hypoxia–ischemia	Inhibition of astrocytes and microglia activation by reducing the expression of inflammatory markers, *viz.*, TNF-α and IL-1β, with an increase in the expression of IL-10 protein	Wu et al. (2017)

### Specific Evidence in Neurodegenerative Models

Alzheimer’s disease (AD) is a chronic neurodegenerative disorder characterized by cognitive and memory impairments (Kumar and Singh 2015). It also results in the formation of neurofibrillary tangles from abnormally phosphorylated tau and abnormal accumulation of amyloid plaques (Drummond and Wisniewski 2017). Animal models play a crucial role in defining disease-associated mechanisms and have been of prime importance in evaluating the effectiveness of novel therapeutic agents (Drummond and Wisniewski 2017). In a rodent model of AD, COS (chitosan oligosaccharides) (200, 400, or 800 mg/kg BW for 2 weeks) were found effective in reducing cognitive deficits in amyloid-β-_1–42_-induced rats. Inhibition of oxidative stress and suppression of inflammatory response *via* the reduction in the levels of IL-1β and TNF-α are reported to influence cognitive functions (Jia et al., 2016). Similar observations were reported for D-galactose AD rat models wherein supplementation of FOS (2.5 and 5% w/w; for 49 days) improved spatial learning and memory by reducing Aβ density in the cortex and hippocampus with improvement in the plasma ascorbic acid level in a dose-dependent manner (Yen et al., 2017). A study by Chen et al. (2017) indicated that FOS from *Gynochthodes officinalis (F.C.How) Razafim. & B.Bremer* exerted memory improvements in adult male Sprague-Dawley rats model of AD. They investigated the role of FOS extract in alleviating symptoms of AD by targeting the microbiota effects of the gutbrain axis. Rats were randomly assigned into 4 groups. The first group served as control; the second group of rats received D-galactose (100 mg/kg BW/day); the third group received low dose of FOS extract (50 mg/kg BW/day) + D-galactose (100 mg/kg BW/day); the fourth group received high dose of FOS extract (100 mg/kg BW/day) + D-galactose (100 mg/kg BW/day). FOS extract administration showed a marked effect on the AD-associated cognitive behavior by improving oxidative stress, enhancing neurotransmitter synthesis (norepinephrine, dopamine, 5-hydroxytryptamine, and 5-hydroxyindole acetic acid) in rats. Furthermore, FOS administration could significantly down-regulate the expression of AD-related intracellular markers (tau and Aβ_1−42_). This effect is attributed to the fact that FOS extract administration could modulate the interaction between gut ecology and brain physiology *via* the gutbrain axis. The disaccharide, lactulose is reported to offer beneficial effects towards AD. Pretreatment of lactulose offered neuroprotection in the mice model by increasing the levels of the autophagic pathways and decreasing neuroinflammation, thereby attenuating short-term memory and the learning retrieval associated with AD (Lee et al., 2021).

A significant number of research findings have demonstrated the link between gut microbiota alterations on the onset of ASD. The beneficial effect of probiotic administration in ASD children is evaluated by many researchers as they are known to restore gut microbiota and downregulate ASD symptoms. Probiotic strains, *viz.*, *Lactobacillus acidophilus* (Liu et al., 2017), *Lactobacillus plantarum* (Shaaban et al., 2018), *Lactobacillus rhamnosus* (Kałużna-Czaplińska and Błaszczyk, 2012), and *Bifidobacterium longum* (Niu et al., 2019) have shown therapeutic potential towards ASD. In line with this, there have been few studies that have examined the use of prebiotics in ASD children (Grimaldi et al., 2018; Sanctuary et al., 2019). Tolerability and efficacy of a probiotic strain (*Bifidobacterium infantis*; 20 billion CFU/d) in combination with prebiotic oligosaccharides (bovine colostrum product) to improve immune functions in ASD children and gastrointestinal co-morbidities were evaluated (Sanctuary et al., 2019). Children (9 no.) in the age group 2–11 with a history of frequent gastrointestinal discomfort (constipation, diarrhea, and IBS) were evaluated in the study (12 weeks). The study period comprised of pro-prebiotic supplementation for 5 weeks, followed by a wash-out period (2 weeks) and prebiotic supplementation (5 weeks). Pro-prebiotic supplementation was found to be well tolerated as assessed using the validated questionaries on pediatric gastrointestinal symptoms. Reduction in gastrointestinal symptoms is attributed to the reduction in IL-13 and TNF-α levels after supplementation. Community-level analyses were performed to study how supplementation affects gut microbiome enterotypes. It was found that few participants shifted from *Prevotella* enterotype levels to high *Bifidobacterium* enterotype levels. However, a detailed analysis of the enterotype data revealed that the treatment showed an inconsistent effect on enterotype levels. Grimaldi et al. (2018) studied the effect of exclusion diets (gluten and casein-free diets) and the impact of GOS (6 weeks) on gut microbiota and metabolism in ASD children (30 no.; age group: 5–10). Combining GOS supplementation with an exclusion diet resulted in a significant reduction in gastrointestinal discomfort and anti-sociability scores. Prior to this, the same group of researchers had validated the ability of GOS to alter gut community positively (increased bifidobacterial populations with enhanced levels of SCFA) in autistic children (Grimaldi et al., 2017).

Neonatal hypoxic–ischemic brain injury is a leading cause of neurodevelopmental disabilities in infants. Few research studies have demonstrated the fact that HDAC inhibitors play a beneficial role in adult ischemia models (Fleiss et al., 2012; Ziemka-Nalecz et al., 2017). Ziemka-Nalecz et al. (2017) evaluated the neuroprotective potential of sodium butyrate (as an HDAC inhibitor) in the dentate gyrus of hypoxic–ischemic-injured immature rats. Administration of 300 mg/kg BW sodium butyrate (immediately starting after hypoxic exposure; for 5 consecutive days) resulted in the stimulation of oligodendrocyte precursor cell proliferation in the hippocampal dentate gyrus with a reduction in the microglial cell numbers in the rat ipsilateral hemisphere and enhancement in the BDNF levels in the ipsilateral hemisphere after hypoxic–ischemic brain injury. All these observed parameters demonstrated the neuroprotective effect of sodium butyrate treatment in neonatal rats subjected to hypoxiaischemia. The underlying mechanism for sodium-butyrate-induced HDAC inhibition was explored using the neonatal rat model of hypoxia–ischemia (Jaworska et al., 2019). Wistar rats (7 days old) received sodium butyrate at a dose of 300 mg/kg BW for consecutive 5 days, immediately after hypoxic exposure. The neuroprotective effect of sodium butyrate in hypoxiaischemia is attributed to the neurogenic effect associated with increased BDNF levels with enhanced activation of the TrkB receptor (BDNF receptor) and the phosphorylation of the transcription factor CREB in the ipsilateral hemisphere. This study suggests that BDNF-TrkB signaling plays a vital role in sodium butyrate-induced neurogenesis after hypoxiaischemia. The protective effect of prebiotic NDO, COS (0.1 ml/10 g BW, injected for 2 consecutive days every 12 h after hypoxic exposure) in neonatal hypoxicischemic brain damage was evaluated using Sprague-Dawley rats (7 days) (Wu et al., 2017). Posttreatment with COS resulted in the upregulation of antioxidant enzymes (GSH-PX, SOD, and T-AOC) and downregulation of lactic acid, MPO, and MDA levels in ischemic hemispheres. Furthermore, COS treatment also inhibited astrocytes and microglia activation by reducing the expression of inflammatory markers, *viz.*, TNF-α and IL-1β, with an increase in the expression of IL-10 protein. In conclusion, this study proves the ability of COS as a potential neuroprotective compound against neonatal hypoxicischemic brain damage.

## Current Limitations and Future Perspective

Prebiotic NDOs are a broad category of beneficial compounds supporting good overall nutrition that provide health benefits by stimulating the growth of beneficial microorganisms (probiotics). They support the carbon-based energy needs of intestinally residing probiotics which results in products of metabolism such as SCFAs that get released into the bloodstream. These SCFAs are reported to have a beneficial effect on the GI tract and other distal organs, *viz.*, the brain. Minute quantities of prebiotics that naturally occur in food may not be effective in conferring health benefits. Therefore, strategies are being advanced to produce prebiotics on an industrial scale and incorporate them into designed food and supplement products for improved health benefits. SCFAs produced through the process of bacterial fermentation of prebiotics in the GI tract are hypothesized to confer neuroprotection and sustain mental health by modulating the physiology of the gutbrain axis through various neuro-immunological pathways. Studies have suggested that the gut–brain axis which links and aligns the CNS and ENS, corresponds to a key bidirectional pathway in conferring neuroprotection. The ability of prebiotics to regulate CNS processes through direct and indirect mechanisms by normalizing the gut microbiota, and they offer beneficial effect against various disorders including mental health which ultimately shape cognitive behavior and function. Research advances to date using animal models, though neural (vagus nerve, BDNF, neurotransmitters), endocrine (HPA axis and associated hormones), and immune (immune cells and markers, *viz.*, TNF-α and IP-10) pathways, have been identified to be associated with prebiotic NDOs gutbrain communication. There is an essential need for other mechanistic pathways to be explored. SCFAs influence brain function by inhibiting HDAC activity and enhancing BDNF levels. The available literature has indicated butyrate to be an HDAC inhibitor, and other SCFA, *viz.*, propionic acid when administered at a larger dose has shown autistic (ASD)-like symptoms. Thus, in-depth research studies are required to evaluate the potential use of SCFAs to treat ASD. A thorough understanding of the functional effects of prebiotics and their metabolites (SCFAs) in gutbrain interactions would support the design and development of novel prebiotic therapeutic targets for treating various neurological ailments. A limited number of publications support the fact that peptidoglycan, another prebiotic fermentation product, offers health benefits by influencing the innate immune system against host pathogens (Clarke et al., 2010). However, in-depth studies are required to evaluate the beneficial role of this metabolite. Even though there is a long history of safe consumption of prebiotics, more research is needed on the safety and toxicity considerations of various prebiotic NDOs, especially as novel prebiotics emerge in the market. FOS (> 20 g/day) are reported to enhance the fecal output with flatulence, bloating, abdominal pain, cramps, and diarrhea as side effects. However, these side effects are unlikely to occur when FOS administered is below 20 g/day (Serra et al., 2019). Studies exploring 90 days oral toxicity using animal model found that GOS up to 5,000 mg/kg BW/d resulted in a decrease in food consumption (7–13%) with no significant adverse toxicological effects attributed to clinical pathologies (blood biochemistries, hematology, coagulation, and urinalysis) (Anthony et al., 2006). A single dose of XOS (5,000 mg/kg BW) was found to be well-tolerated and non-toxic in the acute oral toxicity studies (Boonchuay et al., 2021). In line with this, tolerability and toxicity studies for other prebiotics are still underway. Despite promising preclinical findings, prebiotics have demonstrated limited efficacy in the management of behavioral symptoms at the clinical level. Preliminary evidence available at the clinical level supports the fact that prebiotic NDOs are capable of improving brain function and behavior. However, the studies are mainly of short duration (4–12 weeks) and are limited to healthy, young, and middle-aged adults. More research is needed to identify safe and effective dose, delivery method, and duration of application particularly among diseased adults and the geriatric population. Even though early life intake of various prebiotic NDOs is validated to support neurodevelopmental activities at the preclinical level, however, at the clinical level, the results seem to be detrimental. Thus, more reproducible and rigorous research is needed to evaluate early life intake of prebiotic NDOs on neurodevelopmental outcomes to draw an accurate conclusion. Finally, the available preclinical and clinical studies advance the potential application of prebiotics and/or probiotics and their combination as therapeutics in the treatment of brain disorders. Easier production, formulation procedures, and storage advantages in comparison to probiotics make prebiotics promising candidates for promoting better health. Further research on effective formulation with clinical studies is essential to advance the important potential of prebiotics to improve brain health and wellness and advance them as essential therapeutic candidates to incorporate in dietary and nutraceutical formulations.
